# VATS intraoperative tattooing to facilitate solitary pulmonary nodule resection

**DOI:** 10.1186/1749-8090-3-13

**Published:** 2008-03-19

**Authors:** Lourens Willekes, Cherif Boutros, Michael A Goldfarb

**Affiliations:** 1 Department of Surgery, Monmouth Medical Center, NJ, USA

## Abstract

**Introduction:**

Video-assisted thoracic surgery (VATS) has become routine and widely accepted for the removal of solitary pulmonary nodules of unknown etiology. Thoracosopic techniques continue to evolve with better instruments, robotic applications, and increased patient acceptance and awareness.

Several techniques have been described to localize peripheral pulmonary nodules, including pre-operative CT-guided tattooing with methylene blue, CT scan guided spiral/hook wire placement, and transthoracic ultrasound.

As pulmonary surgeons well know, the lung and visceral pleura may appear featureless on top of a pulmonary nodule.

**Case description:**

This paper presents a rapid, direct and inexpensive approach to peripheral lung lesion resection by marking the lung parenchyma on top of the nodule using direct methylene blue injection.

**Methods:**

In two patients with peripherally located lung nodules (n = 3) scheduled for VATS, we used direct methylene blue injection for intraoperative localization of the pulmonary nodule. Our technique was the following: After finger palpation of the lung, a spinal 25 gauge needle was inserted through an existing port and 0.1 ml of methylene blue was used to tattoo the pleura perpendicular to the localized nodule.

The methylene blue tattoo immediately marks the lung surface over the nodule. The surgeon avoids repeated finger palpation, while lining up stapler, graspers and camera, because of the visible tattoo. Our technique eliminates regrasping and repalpating the lung once again to identify a non marked lesion.

**Results:**

Three lung nodules were resected in two patients. Once each lesion was palpated it was marked, and the area was resected with security of accurate localization. All lung nodules were resected in totality with normal lung parenchymal margins. Our technique added about one minute to the operative time. The two patients were discharged home on the second postoperative day, with no morbidity.

**Conclusion:**

VATS with intraoperative tattooing is a safe, easy, and accurate technique to streamline and efficiently resect solitary pulmonary nodules.

## Introduction

Video-assisted thoracic surgery (VATS) has been applied over the last decade to virtually every disease process encountered in thoracic medicine [1]. Its use as a diagnostic tool in pleural and pulmonary disease is well established especially in evaluating solitary pulmonary nodules, and pulmonary processes of unclear etiology.

VATS has significantly changed the management of patients with solitary pulmonary nodule. To avoid thoracotomy, many patients undergo trans-thoracic needle biopsy in an attempt to establish a diagnosis. However, the absence of findings of malignancy on pathologic examination of trans-thoracic needle specimen is not sufficient to prove that a solitary pulmonary nodule is benign. As high as 29% of patients whose condition was not diagnosed as malignant on trans-thoracic needle biopsy were ultimately found to have carcinoma [2] Since VATS wedge excision, which can be performed with a hospital stay of 24 to 48 hours and minimal postoperative disability, trans-thoracic needle biopsy is used less often.

Currently when performing a VATS lung biopsy for solitary pulmonary nodule, a finger is inserted through one of the port sites to palpate the lung and find the lesion. Usually, the majority of the lung can be felt in this manner, with an instrument placed through another port site to move the lung toward the examining finger. Suzuki and associates in a series of 92 consecutive patients undergoing VATS for pulmonary nodule reported conversion to thoracotomy in fifty patients (54%). The most common reason for the conversion was failure to localize nodules (46%) [3].

Despite other techniques developed to localize pulmonary nodules, small nodules with no surface landmarks remain a challenge. Often the surgeon regrasps, and repalpates the lung multiple times to be sure of the accurate localization of the lesion. The featureless visceral pleura may have no puckering or discoloration to give clues to nodule location.

## Case description

We developed another technique for intraoperative marking of lung nodules. Our technique involves tattooing the lung parenchyma overlying the palpated lung lesion with methylene blue injected on the surface of the lesion through one of the port sites. This tattoo is then used as landmark for the biopsy site.

## Methods

### First Case

**Figure 1:**
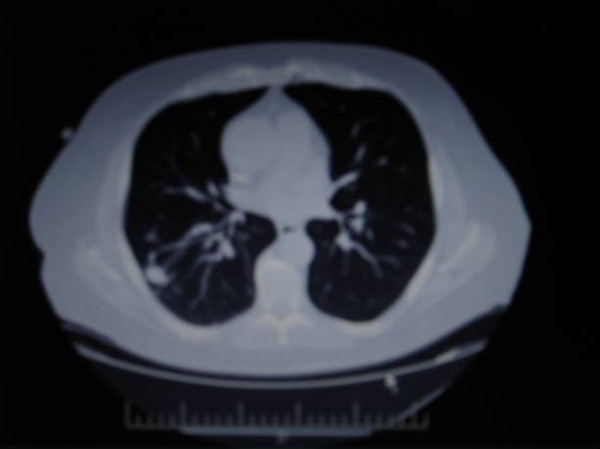
**Chest CT scan.** Nodule involving the right lower lobe, laterally, measures 1.1 × .6 cm.

**Figure 2:**
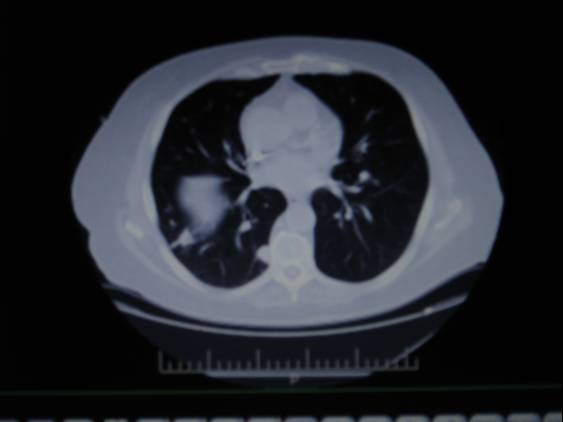
**Chest CT scan.** Nodule involving the right lower lobe, medially, measuring approximately 1.2 × 1 cm, seen adjacent to the thoracic spine

**Figure 3:**
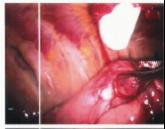
Lateral lung nodule detected by finger palpation through the superior port site.

**Figure 4:**
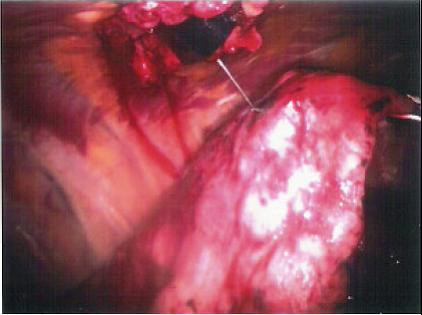
Lateral lung nodule marking by injection of 0.1 mL methylene blue through the superior port site.

**Figure 5:**
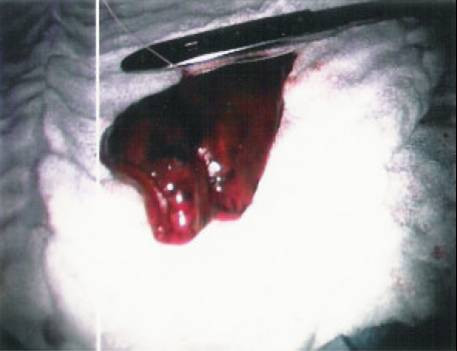
The lateral lung nodule marked area extracted.

**Figure 6:**
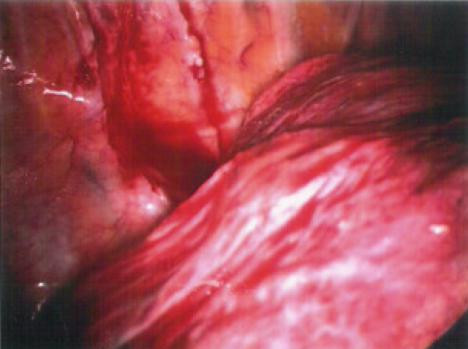
Finger palpation of the posteromedial lung nodule.

**Figure 7:**
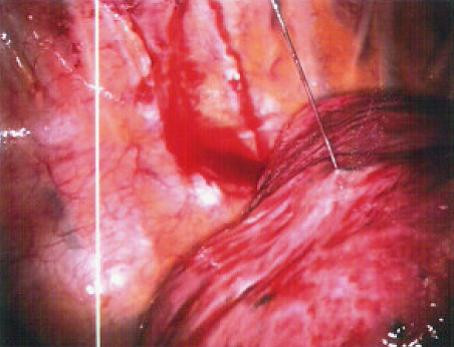
Injection of 0.1 mL of methylene blue marking the area of the posteromedial lung lesion.

**Figure 8:**
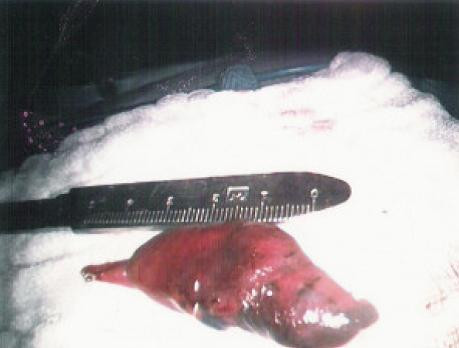
The posteromedial lung nodule marked area extracted.

The patient is a 66-year-old white male with a past history of end-stage renal disease, hypertension, hyperlipidemia, diabetes, gastroesophageal reflux disease, and anemia. He had two pulmonary nodules on a CT scan of the chest measuring 1.5 and 1.8 cm. A PET scan was performed and showed a high uptake in area of these nodules (maximum SUV 5.2,6.1 respectively). The patient denied tobacco or alcohol use. He had no significant occupational environment exposure. A repeat CT scan three months later showed a decrease in size of the pulmonary nodules (1.1, 1.2 cm) but their etiology remained uncertain (Figures 1, 2). Under general anesthesia with a double-lumen endotracheal tube, the patient was positioned with the right side up. Three 1.5-cm ports were placed in a triangular fashion around the tip of the scapula. The 30-degree 10-mm viewing scope was inserted. The first lesion was palpated (Figure 3). In order to localize the lesion under a featureless lung surface, we injected 0.1 ml of methylene blue dye just underneath the visceral pleura using a spinal needle 25 Gauge through the posterior port under camera guidance (Figure 4). The camera and the operating instruments were then placed in ideal position and exchanged so as to provide a good angle for the stapling device to completely wedge out the lesion. The EZ45/4.8 standard stapling device was utilized to perform a wedge resection of the palpable abnormality (Figure 5). Attention was then turned to the posterior basal segment of the right lower lobe where the second lesion was palpated (Figure 6). After palpation, the area was marked with 0.1 ml of subpleural injection of methylene blue through the port as previously described (figure 7). The tattoo allowed us to remove the camera and exchanged grasper and camera positions and easily identify the lesion upon reentry. The lesion was biopsied in a similar fashion (Figure 8). Both lesions were removed using an EndoCatch bag and sent for frozen section

### Second case

**Figure 9:**
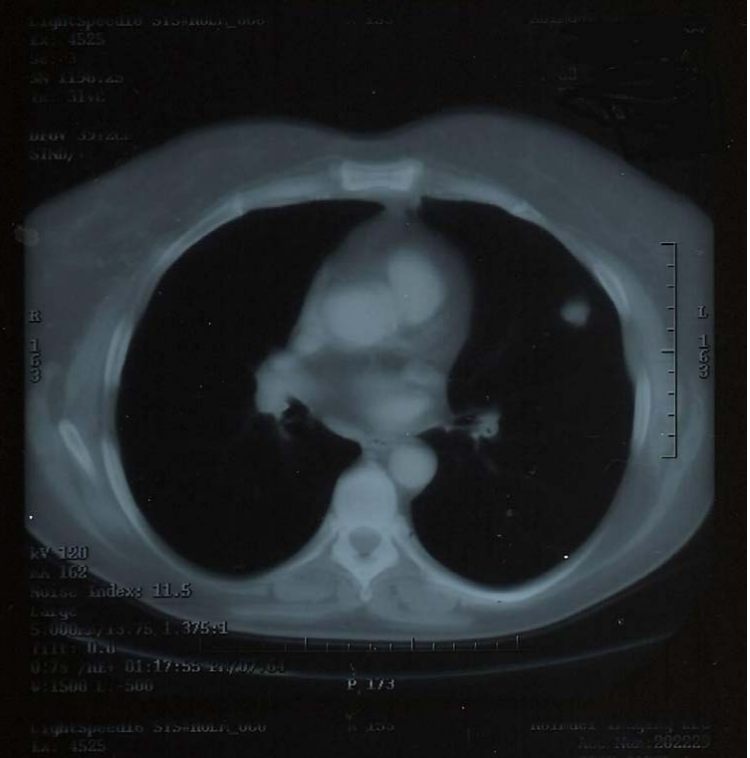
**Chest CT scan.** Left upper lobe pulmonary nodule.

**Figure 10:**
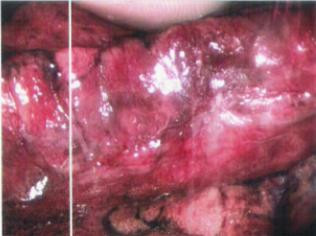
Finger palpation of the left upper lobe lung nodule.

**Figure 11:**
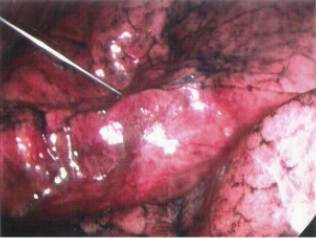
Methylene blue tattooing of the palpated lesion.

The second patient is a 63-year-old white female with no significant past medical history. She was a former heavy smoker, having quit 14 years ago. She had an incidentally discovered lung nodule on a chest x ray that was ordered preoperatively for an elective surgery. A chest CT showed a well circumscribed solitary nodule in the left upper lobe measuring 1.5 cm (Figure 9). The patient had general endotracheal anesthesia with a double-lumen tube, and was placed in the lateral position with the left side up. Three 1.5-cm ports were positioned in a triangular fashion around the tip of the scapula. Using 30-degree camera, examination of the left lung by finger palpation revealed a slightly firm area in the left upper lobe (lingular portion) that was approximately 2 cm in dimension (Figure 10). The lesion was marked with 0.1 mL of methylene blue dye just underneath the visceral pleura over the lesion (Figure 11). The camera and the working ports were exchanged and an EZ 45/4.8 standard stapler was then used to perform generous wedge resection of the tattooed area. The entire specimen was retrieved from the chest cavity in an EndoCatch bag.

## Results

These cases had no perioperative morbidity. The tattooing technique added minimal time to the surgical procedures.

Frozen section for both lesions of the first case revealed no malignancy. The final pathology report revealed granulomatous pneumonitis (fungal infection) in both nodules with complete excision. The fungal culture revealed histoplasma capsulatum. The nodules measured 2 × 1 × 1 cm and 0.7 × 0.5 × 0.5 cm. The patient had an uneventful postoperative course and was discharged from the hospital on the second postoperative day.

With regard to the second case, frozen section revealed squamous cell carcinoma with the whole lesion excised and 2 cm margins. A lobectomy was precluded as her preoperative pulmonary test showed an FEV of 0.62 L/sec. Final pathology revealed that the nodule measured 1.5 × 1 × 1 cm and was found to be 0.1 cm from the pleural surface. Mediastinal lymph nodes were sampled and were tumor free. The patient had an uneventful postoperative course and was discharged on the second postoperative day.

## Discussion

Video-assisted thoracic surgery (VATS) is an excellent procedure for diagnosis and treatment of peripheral pulmonary nodules. However, thoracoscopy has limits in the detection of small nodules, below the pleural surface, deep in the lung parenchyma, which cannot be seen as much as palpated

The nodule is often located based on anatomical landmarks, pre-operative CT scan, and finger palpation. Also, there may be changes in the appearance of the overlying visceral pleura (puckering, discoloration, etc.) which make the localization easier.

There are many pulmonary nodules, however, which have no obvious landmark for subsequent extraction. The lung surface is often featureless and the nodule frequently "disappears" after finger palpation.

Furthermore, the surgeon may have to change the camera, graspers, and stapler positions to optimize access to the nodule. In this process the nodule may be "lost" and must once again be palpated.

Methods to localize such lesions, including the preoperative CT scan guided methylene blue injection, needle localization, and intraoperative ultrasound localization have been used in the past.

The feasibility and effectiveness of the percutaneous CT-guided placement of hook-wires to localize such nodules before video-assisted thoracoscopy was studied by Poretti and associates in 19 patients [4]. They found that the hook was not close to the nodules in four patients, eight patients developed an asymptomatic pneumothorax, and four patients had local bleeding.

The value of preoperative CT scan guided localization of the lung nodule by methylene blue was studied in fifteen patients by Lenglinner and associates [5]. In three cases, pulmonary hemorrhage occurred as a complication of the percutaneous staining procedure. In one case, subsequent conversion to thoracotomy was necessary owing to pulmonary hemorrhage and additional pleural bleeding during VATS. Pneumothorax occurred in five patients.

Another modality used to localize lung nodules is the intraoperative ultrasound. Santambrogio and associates in eighteen patients evaluated the use of a dedicated intraoperative ultrasound probe as an aid in localization of small pulmonary nodules during VATS [6]. They reported success in localizing deep pulmonary nodules less than 20 mm in diameter in all their patients. This technique though effective is more complicated and time consuming than tattooing. Also, after localizing the nodule by ultrasound, actual resection of this grossly invisible nodule will require multiple applications of the ultrasound, to be sure that the surgeon accurately resects the involved lung tissue. This technique, however, is useful when a nodule cannot be palpated.

In our technique, intraoperative tattooing of the lung lesion was performed as soon as the lesion was palpated. A limitation of this technique is the surgeon's ability to palpate the lesion. The smallest lesion that we resected was 0.7 cm. As smaller nodules may not be palpated, perhaps a combination of ultrasound detection and methylene blue tattooing might be an option for these tiny nodules.

Since we performed intraoperative marking with resection of the marked lung tissue, pneumothorax and bleeding were avoided. The injection was performed with camera control and no complications.

Since the injection was through a protected port site, question of dispersing malignant cells through a separate tract, such as through a CT scan guided percutaneous modality, is eliminated.

Any thoracic surgeon should be able to immediately adopt this technique to allow efficient VATS nodule resection.

## Conclusion

VATS intraoperative tattoo is a safe, easy, and accurate technique to streamline and efficiently resect solitary pulmonary nodules.

## Competing interests

The author(s) declare that they have no competing interests.

## Authors' contributions

LW carried out the study conception and design, and helped in drafting the manuscript. CB carried out data acquisition, drafting the manuscript and helped in the study conception and design. MG carried out the study conception and design as well as the critical revision of the manuscript. All authors read and approved the final manuscript.
